# A Glycolipid α-GalCer Derivative, 7DW8-5 as a Novel Mucosal Adjuvant for the Split Inactivated Influenza Vaccine

**DOI:** 10.3390/v14061174

**Published:** 2022-05-28

**Authors:** Huapeng Feng, Ruolin Sun, Guanru Song, Shunfan Zhu, Zhenyu Nie, Liming Lin, Ruonan Yi, Shixiang Wu, Genzhu Wang, Yulong He, Siquan Wang, Pei Wang, Li Wu, Jianhong Shu

**Affiliations:** 1 Department of Biopharmacy, College of Life Sciences and Medicine, Zhejiang Sci-Tech University, Hangzhou 310018, China; srl991020@163.com (R.S.); sxsongguanru@126.com (G.S.); zhushunfan2022@163.com (S.Z.); nzyzs1997@163.com (Z.N.); llm980102@163.com (L.L.); yirn1128@163.com (R.Y.); wsxzl0725@163.com (S.W.); wanggenzhu1232021@163.com (G.W.); heyulong2003@163.com (Y.H.); w2584313659@163.com (S.W.); w2621373868@163.com (P.W.); 2 Department of Biology, College of Life Sciences, China Jiliang University, Hangzhou 310018, China

**Keywords:** glycolipid, 7DW8-5, mucosal adjuvant, influenza vaccine

## Abstract

Influenza virus infects the host and transmits through the respiratory tract (i.e., the mouth and nose); therefore, the development of intranasal influenza vaccines that mimic the natural infection, coupled with an efficient mucosal adjuvant, is an attractive alternative to current parenteral vaccines. However, with the withdrawal of cholera toxin and Escherichia coli heat-labile endotoxin from clinical use due to side effects, there are no approved adjuvants for intranasal vaccines. Therefore, safe and effective mucosal adjuvants are urgently needed. Previously, we reported that one derivative of α-Galactosylceramide (α-GalCer), 7DW8-5, could enhance the protective efficacy of split influenza vaccine by injection administration. However, the mucosal adjuvanticity of 7DW8-5 is still unclear. In this study, we found that 7DW8-5 promotes the production of secret IgA antibodies and IgG antibodies and enhances the protective efficacy of the split influenza vaccine by intranasal administration. Furthermore, co-administration of 7DW8-5 with the split influenza vaccine significantly reduces the virus shedding in the upper and lower respiratory tract after lethal challenge. Our results demonstrate that 7DW8-5 is a novel mucosal adjuvant for the split influenza vaccine.

## 1. Introduction

Vaccination is an effective strategy to control influenza. Currently, there are two types of seasonal influenza vaccines for human use: inactivated influenza virus vaccines, including the whole-virus inactivated influenza vaccine, influenza virus split vaccine, and influenza virus subunit vaccine (e.g., FluBlok^®^); and live attenuated influenza virus vaccine (e.g., FluMist^®^). The seasonal influenza vaccines most frequently administered to humans are the split and subunit vaccines, which possess high safety profiles, but show weak immunogenicity, especially among children and the elderly [1,2]. Moreover, these vaccines cannot induce secreted IgA or T-cell immunity, so they do not induce significant cross-protection [3], and they must be injected, which is a disincentive to some individuals. Live attenuated influenza virus vaccines can induce the production of secretory IgA and cellular immunity. Still, the vaccine effectiveness may be affected by anti-influenza virus antibodies already present in the recipient [1,4]. In addition, due to safety concerns, the live attenuated vaccine cannot be administered to children under the age of 2 years, adults 50 years and older, people with weakened immune systems, and pregnant women [5] (https://www.cdc.gov/flu/prevent/nasalspray.htm, accessed on 1 March 2022). Therefore, more effective influenza virus vaccines are needed.

The influenza virus infects mainly through the upper respiratory tract; therefore, the establishment of mucosal immunity in the upper respiratory tract is an attractive strategy to control influenza. Nasal delivery of inactivated influenza vaccine is an attractive alternative because it can induce both the mucosal immune response (secretory IgA antibodies) and the systemic immune response (IgG antibodies) [5,6]. Moreover, nasal-delivery inactivated influenza vaccine can provide cross-protection against a mismatched circulating virus due to the induction of secretory IgA antibodies [7,8]. Since the inactivated influenza vaccine per se cannot induce strong mucosal and systemic immunity due to the harsh mucosal environment and weak immunogenicity, an effective mucosal adjuvant must be formulated with the inactivated influenza vaccine, especially a recent split vaccine [9]. Subunit B of cholera toxin or heat-labile enterotoxin was used as a mucosal adjuvant for a split influenza vaccine, but was withdrawn from clinical use due to an increased risk of Bell’s palsy [10,11]. Currently, there are no approved adjuvants for inclusion in licensed mucosal vaccines.

7DW8-5, a synthetic glycolipid derived from the α-galactosylceramide (α-GalCer), is a CD1d-dependent iNKT cell agonist [12]. Its adjuvant effect is appropriately 100 times that of α-GalCer [13]. Previously, 7DW8-5 has shown an adjuvant effect on adenovirus-vectored malaria vaccines by intramuscular injection [14]. Previously, we reported that 7DW8-5 enhances the protective effect of the split seasonal influenza vaccine by parental administration in mice [15]. Moreover, the α-GalCer improves the humoral and cellular responses induced by inactivated PR8 virus antigen and HIV peptide antigen in mice with intranasal inoculation [16,17]. The alpha-C-galactosylceramide (alpha-C-GalCer), one analog of α-GalCer, enhanced the protective efficacy and reduced the amount required for the protection of live attenuated influenza vaccine by intranasal immunization [18]. However, the effect of 7DW8-5 as a mucosal adjuvant is still unknown.

In this study, we evaluated the adjuvant effect of 7DW8-5 on the split inactivated influenza vaccine by intranasal immunization and found that 7DW8-5 is a good mucosal adjuvant candidate for the seasonal split inactivated influenza vaccine.

## 2. Materials and Methods

### 2.1. Ethic Statement

All experiments with mice were performed in accordance with the Regulations for Animal Care and the Guidelines for Proper Conduct of Animal Experiments by the Zhejiang Sci-Tech University. All of the experiments were approved by the Experimental Animal Welfare Ethics Committee of the College of Life Sciences and Medicine, Zhejiang Sci-Tech University.

### 2.2. Cells and Viruses

Madin–Darby canine kidney (MDCK) cells (Cat No: CCL-34) were purchased from American Type Culture Collection and were cultured in minimum essential medium (MEM) (Gibco) supplemented with 5% newborn calf serum (AusgeneX, Queensland, Australia) at 37 °C in 5% CO_2_. MDCK cells were used to detect the viral amount in the nasal wash and lungs of the challenged mice.

Mouse-adapted A/California/04/2009 virus (H1N1; MA-CA04) was generated in our laboratory by reverse genetics as previously described [19,20], and was used to evaluate the protective efficacy of the influenza vaccine. Inactivated and purified A/California/07/2009 virus (H1N1; CA07) is one of the components of the commercial split influenza HA vaccine, was used as an antigen for the ELISA to determine the virus-specific antibody titers of samples collected from the immunized mice.

### 2.3. Influenza Vaccines and Compound Preparation Used for the Evaluation

The split influenza hemagglutinin (HA) vaccines were manufactured by DENKA SEIKEN Co., Ltd. (Tokyo, Japan). One of the components of the commercial influenza HA vaccine for the 2016–2017 seasons, the HA proteins from CA07 (H1N1) was used as target antigen for the evaluation of adjuvant effect of 7DW8-5 on the inactivated influenza vaccine. Poly(I:C) (GE Healthcare, Chicago, IL, USA) was used as a positive control as described previously [21].

The 7DW8-5 powder was first dissolved into PBS containing 10% Tween-80 at 10 mg/mL. 7DW8-5 suspension was heated up at 57 °C for 5 min, followed by sonicating it in a water bath for 5 min, then, it was cooled down and aliquoted after mixing well. The aliquots were stored at −20 °C as stocks until use. The solutions were heated up and were sonicated for 5min again before mixing with HA antigen. The solution of 7DW8-5 was incubated with HA influenza vaccine at room temperature with agitation before the immunization.

### 2.4. Immunization and Challenge

Five-week-old female BALB/c mice were purchased from Shanghai SLAC Inc. After one week of adaptation, and the mice were immunized with influenza HA vaccine (the 2016–2017 season) [(0.1 µg/dose in 20 µL with 10 µL/nostril) calculated on the basis of the amount of HA from CA07 (H1N1)] with or without compounds (1 or 10 µg/dose) via intranasal administration under anesthesia by isoflurane. The mice were immunized intranasally twice with a two-week interval between vaccinations. Two weeks after the second immunization, blood was collected via the facial vein by using a goldenrod animal lancet (5 mm), and sera were isolated to measure virus-specific IgG antibody titers. Three weeks after the second immunization, the immunized mice were challenged intranasally, under anesthesia by use of isoflurane, with 10 MLD_50_ (dose required to kill 50% of infected mice; 50 µL/mouse with 25 µL/nostril) of MA-CA04 virus. Body weight and survival were monitored daily for 14 days after virus challenge. Mice which lost more than 25% of their original body weight were euthanized. Four mice per group were used in the protective efficacy evaluation.

For IgA antibody titers and virus replication tests, the mice were immunized intranasally twice with a two-week interval between vaccinations. On day 14 after the second immunization, nasal washes were obtained from the nasopharynx by flushing the nasal passage with 1 mL of PBS, and bronchoalveolar lavage fluid (BALF) was collected by washing the organs first with 0.7 mL of PBS and then with 0.5 mL of PBS as described previously [22]. For the virus replication test, two weeks after the second immunization, the immunized mice were challenged intranasally, under anesthesia, with 10 MLD_50_ of MA-CA04 virus. To determine virus replication in mice, nasal wash and BALF were harvested as described above on days 3 and 6 post challenge; the viral load in NWs and BALFs were determined by using a plaque assay on MDCK cells as described previously [23].

### 2.5. Measurement of Virus-Specific Antibody Titers

Virus-specific antibody titers in nasal wash, BALF, and sera were determined by using a modified ELISA as previously described [24,25]. Briefly, 96-well ELISA plates were coated with 6 µg/mL of inactivated and purified CA07 virus solution overnight at 4 °C (50 µL/well). The plates were then blocked with 200 µL of 20% Blocking One (Nacalai) diluted in water at room temperature for 1 h. After blocking, the plates were washed once with PBS containing 0.05% Tween-20 (PBS-T), and then 2-fold serially diluted samples were added to the plates, followed by a one-hour incubation at room temperature. Bound IgG was detected by using peroxidase-labeled goat anti-mouse IgG (gamma) antibody, F (ab’) 2 fragment (Kirkegaard & PerryLaboratory Inc., Gaithersburg, MD, USA), and bound IgA was detected by using horseradish peroxidase-conjugated goat anti-mouse IgA antibody (Kirkegaard & PerryLaboratory Inc.). After the plates were washed four times with PBS-T, 100 µL of 2, 2′-azino-bis (3-ethylbenzothiazoline-6-sulphonic acid) diammonium salt substrate solution was added to each well to initiate the color reaction, and the OD was measured at a wavelength of 405 nm. The antibody titer was defined as the reciprocal of the highest serum dilution that produced an OD_405_ >0.1 after correcting for the negative serum control [26].

### 2.6. Statistical Analysis

Values are presented as the mean ± standard deviation (SD). For comparing the significant difference between the two groups, we used the Student *t*-test. For multiple comparisons, one-way or two-way analysis of variance (ANOVA) followed by Dunnett’s test was performed by using GraphPad Prism Version 6.07 (GraphPad Software Inc., San Diego, CA, USA). For the analysis of the survival data, we used the log-rank test, comparing the vaccine plus 7DW8-5 or Poly(I:C) group to the vaccine-alone group; we used the OASIS 2 [27] software for this analysis. *p* < 0.05 was considered to indicate a statistically significant difference.

## 3. Results

### 3.1. 7DW8-5 Signifiantly Increases the Mucosal and Systemic Immunogenicity of the Split Influenza Vaccine by Intranasal Administration

**Figure 1:**
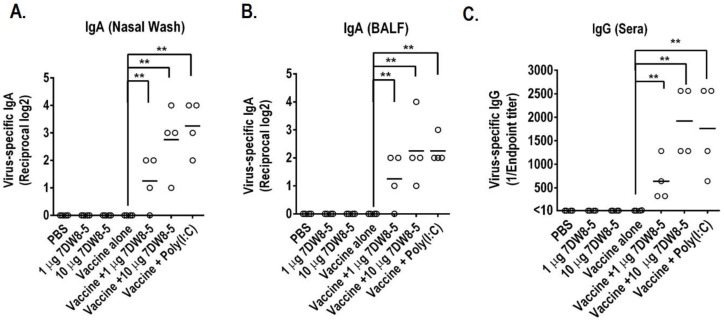
Virus-specific secretory IgA (S-IgA) titers in the respiratory tract and IgG in sera induced by intranasal co-administration of 7DW8-5 with the split inactivated influenza vaccine. Six-week-old BALB/c mice were intranasally immunized with the indicated immunogens (20 μL total, 10 μL/nostril) twice with a two-week interval between immunizations. Four mice were used per group. Nasal wash (NW) and bronchoalveolar lavage fluid (BALF) samples were collected two weeks after the last immunization to measure virus-specific secretory IgA and IgG antibody titers. The virus-specific IgA and IgG titers were determined by use of an ELISA with inactivated CA07 virus as the coating antigen. The OD was measured at a wavelength of 405 nm. The antibody titer was defined as the reciprocal of the highest dilution that produced an OD405 >0.1 after correcting based on the negative serum control. The values are the means ± SD of the four individual antibody titers per group. (**A**) Virus-specific IgA antibody titers in the NWs induced by intranasal co-administration of 7DW8-5 with the split inactivated influenza vaccine. (**B**) Virus-specific secretory IgA antibody titers in the BALFs. (**C**) Virus-specific IgG antibody titers in sera. ** *p* < 0.01.

The mice were immunized twice at a two-week interval, and the nasal wash, BALF, and sera were collected at Day 14 after the second immunization for the IgG and IgA antibody detection. The results show that 1 µg 7DW8-5 or 10 µg 7DW8-5 per dose both significantly increased the titers of the IgA antibodies in the nasal wash and BALF and IgG antibodies in sera compared to the vaccine alone (Figure 1A–C), although the effect of 10 µg 7DW8-5 per dose was better than that of 10 µg 7DW8-5 per dose by intranasal immunization. Furthermore, the 10 µg 7DW8-5 induced a little higher IgG antibody level than that of Poly(I:C) in sera, although these two groups had no significant difference (Figure 1C). These results demonstrate that 7DW8-5 could enhance the mucosal and systemic immune responses of the inactivated influenza vaccine by intranasal administration.

### 3.2. 7DW8-5 Enhances the Protective Efficacy of the Influenza Vaccine against Lethal Challenge

**Figure 2:**
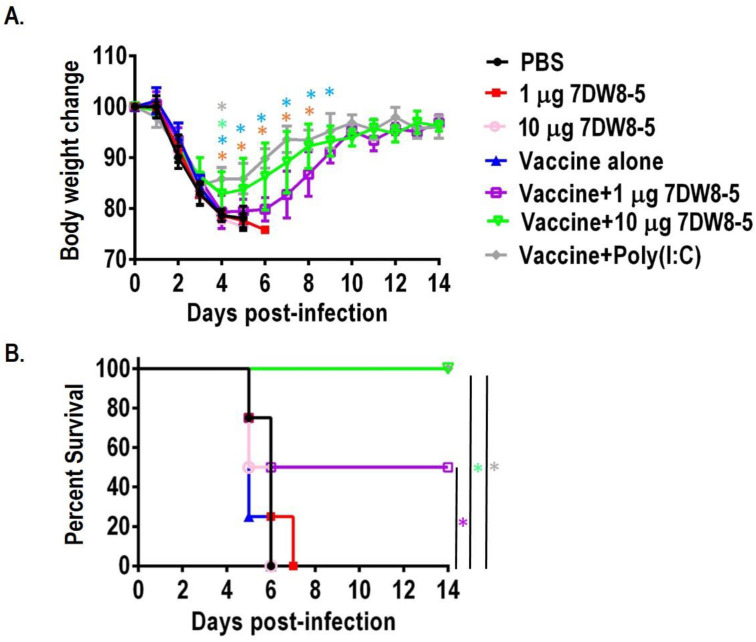
Body weight change and survival rate of the immunized mice with the split inactivated influenza vaccine plus 7DW8-5 by intranasal administration after a lethal challenge by MA-CA04 virus. Six-week-old BALB/c mice were intranasally immunized with the indicated immunogens (20 μL total, 10 μL/nostril) twice with a two-week interval between immunizations. Four mice were used per group. Three weeks after the second immunization, the mice were challenged with 10 MLD_50_ of MA-CA04 virus. Body weight and survival were monitored daily for 14 days. (**A**) Body weight change in the immunized mice after lethal infection; (**B**) Survival of the immunized mice after lethal challenge. Green asterisks indicate a significant difference between the vaccine + 10 µg 7DW8-5 group and the vaccine alone; gray asterisks indicate a significant difference between the vaccine + Poly(I:C) group and vaccine-alone group; purple asterisks indicate a significant difference between the vaccine + 1 µg 7DW8-5 group and vaccine-alone group; orange asterisks indicate a significant difference between the vaccine plus 10 µg of 7DW8-5 group and vaccine + 1 µg 7DW8-5 group; blue asterisks indicate a significant difference between the vaccine + Poly(I:C) group and the vaccine + 1 µg 7DW8-5 group. * *p* < 0.05.

To evaluate the effect of 7DW8-5 on the protective efficacy of the influenza vaccine by intranasal inoculation, we immunized the mice twice with 1 µg or 10 µg per dose 7DW8-5 at a two-week interval; the immunized mice were challenged with 10 MLD50 MA-CA04 virus. The body weight and survival were monitored every day for 14 days. In the PBS group (black line), the 1 µg 7DW8-5 group (red line), the 10 µg 7DW8-5 group (pink line), and the vaccine-alone group (blue line), body weight decreased after virus challenge, and all mice died by day 6 (Figure 2). In contrast, in the vaccine + 10 µg 7DW8-5 group (green line) and vaccine + Poly (I:C) (gray line), all the immunized mice survived after lethal challenge. A total of 50% of the mice immunized with vaccine + 1 µg 7DW8-5 group were protected from lethal infection, and the body weight of the survival mice presented a substantially decreased. The body weight loss of the mice immunized with vaccine plus 10 µg 7DW8-5 or vaccine plus Poly(I:C) after the challenge was also observed, but it was significantly less than that in vaccine plus 1 µg 7DW8-5 group (Figure 2A). These results show that 7DW8-5 could enhance the protective efficacy of the split influenza vaccine by intranasal immunization, and the effect of 7DW8-5 as a mucosal adjuvant is dose-dependent.

### 3.3. 7DW8-5 Restricts the Influenza Virus Replication in the Respiratory Tract of the Mice after Administrating Intranasally with the Vaccine Together

To examine the effect of immunization of mice with HA vaccine + 7DW8-5 on virus replication, mice were intranasally immunized with the indicated immunogens twice with a two-week interval between immunizations and they were then challenged with 10 MLD_50_ of MA-CA04 virus three weeks after the last vaccination. To examine viral replication in the upper and lower respiratory tracts, nasal wash (NW) and BALF samples were collected from the mice after the virus challenge as described in the Materials and Methods section. On day 3 post infection, high virus titers were detected in the NW and BALF samples from all the groups; virus titers in the NW and BALF from the HA vaccine + 10 µg 7DW8-5 or HA vaccine + Poly(I:C)-vaccinated mice were lower than those in the vaccine-alone group; the virus titers in the BALF from the HA vaccine + 1 µg 7DW8-5 group were significantly lower than those in vaccine-alone group (Table 1). On day 6 post infection, the virus in the NWs collected from PBS,1 μg 7DW8-5, 10 μg 7DW8-5, and vaccine-alone groups were detectable and the virus titers were relatively low; however, the virus in at least two of three mice from the HA vaccine + 10 µg 7DW8-5 or HA vaccine + Poly(I:C) was undetectable. The virus titers were significantly lower in the BALF collected from the HA vaccine + 10 µg 7DW8-5 or HA vaccine + Poly(I:C) than those from vaccine-alone group (Table 1). These results suggest that 7DW8-5 enhanced vaccine efficacy by limiting infection at the mucosal compartments in the immunized mice after the virus challenge, consistent with the virus-specific IgA and IgG antibody titer, weight loss, and survival data (Figure 1 and Figure 2).

## 4. Discussion

Many pathogens, including influenza viruses, invade the host through mucosal surfaces [28]. Therefore, developing intranasal vaccines that mimic the natural infection route is an attractive strategy to control influenza. In this study, we evaluate the adjuvant effect of 7DW8-5 on the split inactivated influenza vaccine by intranasal administration. We found that 7DW8-5 enhances the virus-specific IgA in NW and BALF and IgG in sera (Figure 1), increases the survival rate of the mice immunized with vaccine + 7DW8-5 compared to vaccine alone against lethal challenge, and inhibits the virus replication in the upper and lower respiratory tract (Figure 1 and Figure 2 and Table 1). Therefore, our results demonstrate that 7DW8-5 could be a novel adjuvant candidate for an intranasal inactivated influenza vaccine.

Previously, we reported that 7DW8-5 showed an adjuvant effect on the split inactivated influenza vaccine and enhanced the protective efficacy by intramuscular administration (I.M.) [15]. Additionally, we found that intranasal co-administration of 7DW8-5 enhances the virus-specific IgA and increases the survival rate in this study. The mice immunized by intranasal administration presented less weight loss than the mice immunized by I.M., although we used the same amount of 7DW8-5 compound in these two studies (Figure 2) [15]. These different phenotypes may attribute to the induction of IgA by I.N. co-administration of 7DW8-5, which could prevent the virus infection.

The safety of the adjuvant is crucial for its application in vaccines for humans. Although 7DW8-5 is still not yet used in clinical trials, it has been demonstrated that it is very safe to use in the adenovirus-based malaria vaccine in non-human primates even using up to 100 µg by intramuscular administration, and it also was chosen as the clinical adjuvant candidate for the HIV vaccine and malaria vaccine [14]. During our studies, although we did not see a remarkable side effect by I.N. and I.M. co-administration of 7DW8-5 in mice according to observing the injection sites and the health condition, the safety profile of 7DW8-5 by intranasal administration needs to be further studied in the future.

The seasonal split inactivated influenza vaccine mainly contains the HA proteins of influenza viruses, and this type of vaccine is safe, but it possesses weak immunogenicity compared to the whole-inactivated influenza vaccine, especially Th1-biased immune response [29]. Previous studies have shown that 7DW8-5 could enhance the Th1-biased immune response and promote the antibody isotype switch [12,13,15,30]. The adjuvant G3 and lipid nanoparticle improved the Th1 immune response of the split inactivated influenza vaccine by parental injection [31,32]. The vaccine adjuvant chitosan enhanced the antigen-specific Th1 immune response through the STING-cGAS pathway by intranasal inoculation [33]. Therefore, we speculate that 7DW8-5 may enhance the Th1 immune response and antibody isotype switch to promote the protective efficacy of split inactivated influenza vaccine by intranasal administration. We will further explore the cellular immune response induced by 7DW8-5 upon intranasal administration in our further studies.

In summary, we evaluate the adjuvant effect of the glycolipid compound 7DW8-5 on the split inactivated influenza vaccine by intranasal administration in this study. We found that 7DW8-5 promotes the secretory IgA immune response in the respiratory tract and IgG immune response in sera, enhances the protective efficacy of split inactivated influenza vaccine, and reduces the virus shedding in the upper and lower respiratory tract. Furthermore, 7DW8-5 is very safe in non-human primates, and its parental compound KRN7000 has been demonstrated to be well-tolerated in humans in phase I/II clinical trials. Therefore, 7DW8-5 is one novel, promising adjuvant for the intranasal influenza vaccine.

## 5. Conclusions

In this study, we evaluate the mucosal adjuvant effect of one analog of glycolipid α-GalCer, 7DW8-5, on the split inactivated influenza vaccine. We demonstrate that 7DW8-5 enhances the production of the secretory IgA antibodies in the respiratory tract and IgG antibodies and the efficacy of the split inactivated influenza vaccine against lethal infection. Since 7DW8-5 has shown a high safety profile in non-human primates, it is a novel and promising mucosal adjuvant for the split inactivated influenza vaccine.

## Figures and Tables

**Table 1:** Virus replication in the respiratory tract of immunized mice challenged with MA-CA04 virus ^a^.

Immunogen	Mean Virus Titers (Log_10_ PFU/mL) ± SD			
	NW		BALF	
	Day 3 p. i.	Day 6 p. i.	Day 3 p. i.	Day 6 p. i.
PBS	4.5 ± 0.7	1.7 ± 0.7	7.0 ± 0.3	5.9 ± 0.6
1 μg 7DW8-5	4.8 ± 0.4	1.7± 0.4	6.6 ± 0.5	6.2 ± 0.2
10 μg 7DW8-5	5.3 ± 0.8	1.9 ± 0.8	6.8 ± 0.2	5.6 ± 0.7
Vaccine alone	4.1 ± 0.3	2.4 ± 0.6	6.7 ± 0.1	5.4 ± 0.8
Vaccine + 1 μg 7DW8-5	4.3 ± 0.8	1.4, 1.8, ND ^b^	6.1 ± 0.3 *^,c^	4.1 ± 0.6
Vaccine +10 μg 7DW8-5	3.1 ± 0.4 *	ND, 1.0, ND *	6.0 ± 0.1 *	2.9 ± 0.5 *
Vaccine + Poly(I:C)	1.7 ± 1.0 *	ND, ND, ND *	5.3 ± 0.4 *	1.3 ± 0.4 *

^a^ Six-week-old BALB/c mice were immunized with the indicated immunogens (20 μL) twice at a two-week interval and challenged with 10 MLD_50_ of MA-CA04 virus two weeks after the second immunization. The nasal wash (NW) and bronchoalveolar lavage fluid (BALF) were collected from the mice (*n* = 3) on days 3 and 6 post-infection (p.i.), and viral titers were determined in MDCK cells by use of plaque assays. ^b^ ND, not detectable. ^c^,* The *p* value was <0.05 compared with the titer after the challenge of the mice immunized with vaccine alone.
